# Evolutionary dynamics of the LTR-retrotransposon *crapaud* in the *Podospora anserina* species complex and the interaction with repeat-induced point mutations

**DOI:** 10.1186/s13100-023-00311-8

**Published:** 2024-01-13

**Authors:** Ivar Westerberg, S. Lorena Ament-Velásquez, Aaron A. Vogan, Hanna Johannesson

**Affiliations:** 1 Department of Ecology, environmental and Plant Sciences, Stockholm University, Stockholm, 106 91 Sweden; 2 Division of Population Genetics, Department of Zoology, Stockholm University, Stockholm, 106 91 Sweden; 3 Systematic Biology, Department of Organismal Biology, Uppsala University, Norbyvägen 18D, Uppsala, 752 36 Sweden; 4 The Royal Swedish Academy of Sciences, Stockholm, 114 18 Sweden

**Keywords:** Fungi, Transposable elements, Retrotransposons, Genome defense, Classification

## Abstract

**Background:**

The genome of the filamentous ascomycete *Podospora anserina* shows a relatively high abundance of retrotransposons compared to other interspersed repeats. The LTR-retrotransposon family *crapaud* is particularly abundant in the genome, and consists of multiple diverged sequence variations specifically localized in the 5’ half of both long terminal repeats (LTRs). *P. anserina* is part of a recently diverged species-complex, which makes the system ideal to classify the *crapaud* family based on the observed LTR variation and to study the evolutionary dynamics, such as the diversification and bursts of the elements over recent evolutionary time.

**Results:**

We developed a sequence similarity network approach to classify the *crapaud* repeats of seven genomes representing the *P. anserina* species complex into 14 subfamilies. This method does not utilize a consensus sequence, but instead it connects any copies that share enough sequence similarity over a set sequence coverage. Based on phylogenetic analyses, we found that the *crapaud* repeats likely diversified in the ancestor of the complex and have had activity at different time points for different subfamilies. Furthermore, while we hypothesized that the evolution into multiple subfamilies could have been a direct effect of escaping the genome defense system of repeat induced point mutations, we found this not to be the case.

**Conclusions:**

Our study contributes to the development of methods to classify transposable elements in fungi, and also highlights the intricate patterns of retrotransposon evolution over short timescales and under high mutational load caused by nucleotide-altering genome defense.

**Supplementary Information:**

The online version contains supplementary material available at 10.1186/s13100-023-00311-8.

## Background

Transposable elements (TEs) are selfish genetic elements capable of increasing their own copy number within the genome [1]. TE-associated selection, both in the form of an arms-race with the host genome and adaptive insertions [2–4], is assumed to have shaped the evolution of eukaryotic genomes. Fungal genomes are generally relatively small (the average genome size of all fungi is 37.7 Mb [5]) and exhibit lower TE abundances, and fewer TE superfamilies, than plant and animal genomes [6]. However, TEs still play an important role in the evolution of fungal genomes, both in terms of genome size [6–10], and structurally important genomic regions [11]. As a response to proliferation of TEs in fungi there have been multiple fungal-specific host defense systems that have evolved [12], making use of the canonical RNA interference pathway widespread among eukaryotes [13, 14], as well asmethylation [15].

Fungi also possess a specialized defense system called repeat-induced point mutation (RIP) [12, 16–18]. RIP, which was first discovered in *Neurospora crassa* [16], is especially important to consider when studying TEs in fungal genomes where it is present. RIP is active during the sexual cycle and functions by stochastically hypermutating cytosines to thymines in any repeated region of the genome typically above ~ 400 bp [19], although this size limit is not that strict since repeats as small as 150 bp have been observed to be targeted by RIP in *N. crassa* [20]. RIP is context specific, targeting certain dinucleotides more than others, mostly CpA/TpG in *N. crassa* and *Podospora anserina* [21], which belong to the same order, but RIP is likely to function differently in other fungi [22, 23]. RIP mutations can target both repeated copies if they share enough similarity (a minimum of around 80% in *N. crassa* [18]), but the overall shared sequence identity can be significantly lower for RIP to function, if two sequences share short, regularly interspersed regions with high similarity [20, 24].

Classification and curation of TEs is an essential step to decode their roles in genome evolution. The most widely used classification schemes, and most influential in shaping software for classification, are those presented by Wicker et al. and implemented by the database RepBase [25, 26]. In this approach, TEs are classified into a hierarchical system relying on both mode of transposition and similarity. Family level assignment is typically determined by 80% similarity over 80% of the sequence and a total sequence length above 80 bp (“the 80-80-80 rule”) [25]. Subfamily classification thresholds can differ, but are usually based on phylogenetic relationship [25]. However, despite the wide usage of the Wicker/RepBase classification systems, there have been situations when it fails to capture the complexity of TEs and their evolutionary history [27–29]. One example is after ladder-like bursts of subfamily expansion, an evolutionary pattern of many retrotransposons due to their RNA-mediated mechanism of transposition [30–32]. Furthermore for fungi, TE-classification should consider the effect the genome defense system RIP has on TE copies. As RIP mutates any repetitive sequence in the genome, typical TE classification thresholds for family and subfamily classifications that are based on sequence similarity might not reflect the actual relationships of the TEs. The common practice of using a consensus sequence of the copies in a repeat library can cause problems if the copies have been exposed to RIP mutations, and in addition, RIP can also complicate detection of open reading frames (ORFs) and protein domains. In fungi there are no fully developed and widely used classification schemes to tackle the unique challenges of many fungal genomes.

An alternative example of a method for understanding TE family relationships is the use of sequence similarity networks (SSN) [33]. In this study, we explore the use of SSNs to classify LTR-retrotransposons in the species complex of the filamentous ascomycete *Podospora anserina*, and subsequently study their evolution. *P. anserina* has been a model organism for over a 100 years and has been used for studying several molecular biology and genetics-related topics, such as TEs and the genetics of RIP [21, 34–39]. A reference genome of *P. anserina* was first published in 2008 and it contained a full annotation, including a first repeat classification [36]. One of the most abundant repeat families in the genome, a long terminal repeat (LTR) retrotransposon family originally named *crapaud* in Espagne et al. [36] was recently found to exist in several multi-copy variants [39]. LTR retrotransposons have two identical LTRs on each end of the element containing the transcription start site and forming an integral part of the transposition mechanism [40]. In addition, LTR-LTR recombination between the two terminal repeats is common, leaving behind a so-called solo-LTR [41], which have been commonly found in *Podospora* [36, 42]. *P. anserina* is part of a species complex with six other closely related species [38], which all have at least one strain sequenced and assembled into nearly gapless telomere-to-telomere chromosome-level assemblies [39, 43, 44], providing the opportunity to study the dynamics of the LTR retrotransposon *crapaud* over short evolutionary time.

We first used the high-quality genomes of this group of species to thoroughly classify the *crapaud* family based on the variable terminal repeats, using a novel SSN based approach in addition to typical alignment-based manual curation. To then investigate the evolutionary patterns of persistence and expansions of *crapaud* in *Podospora* we utilized a phylogenetic approach. We hypothesized that the variation in the terminal repeats characterize distinct subfamilies that have evolved to escape RIP and have persisted in the *P. anserina* species complex despite this challenge.

## Results and discussion

### The *crapaud* element is the most abundant TE in all but one of the seven species in the *Podospora anserina *species complex

**Fig. 1:**
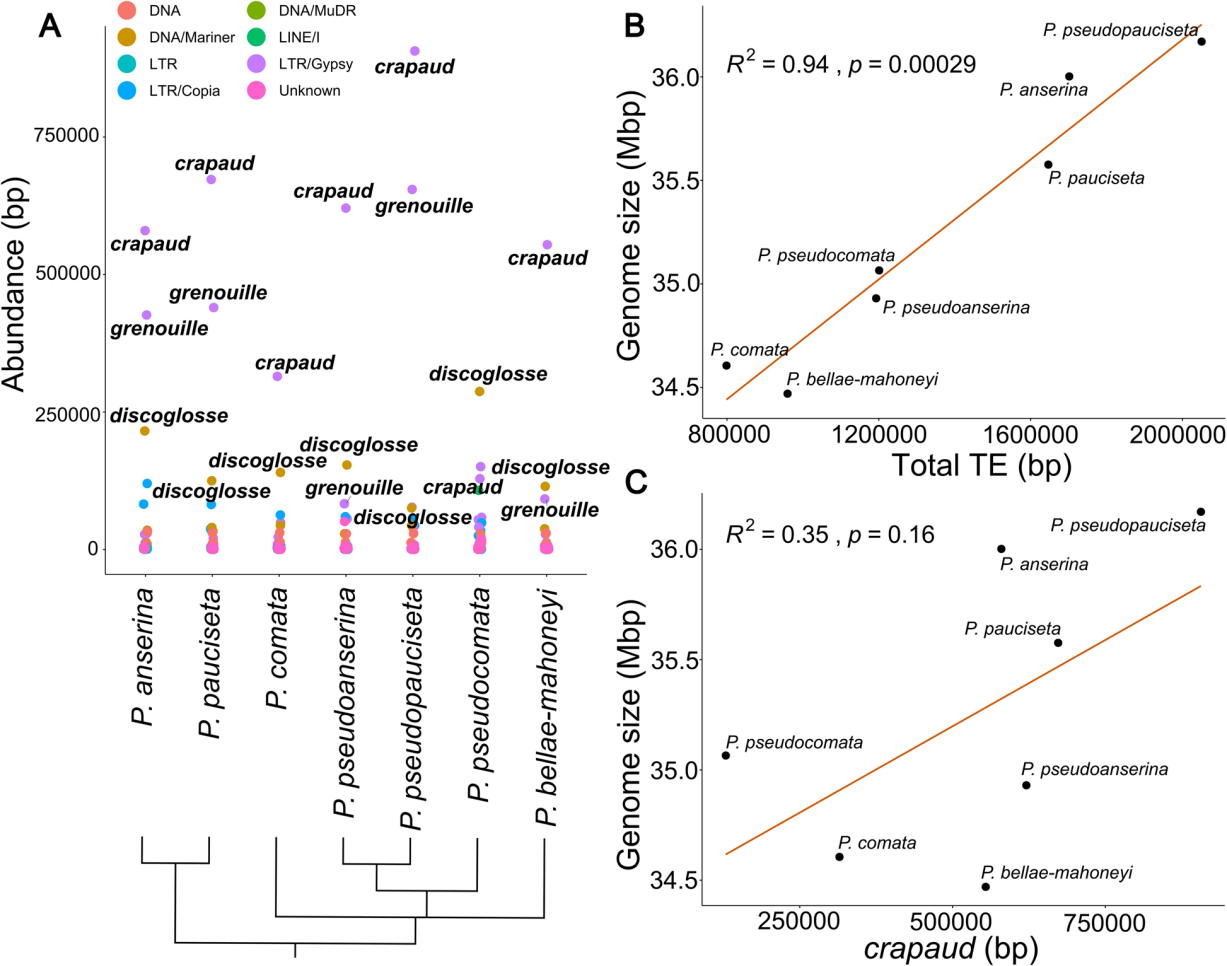
Genomic contributions of transposable element families in the *Podospora anserina* species complex. **A** Base pair abundances of TE families in the *P. anserina* species complex. Colors of points indicate superfamily classification. Names of TE families with an abundance of > 50,000 bp are shown.The relationships between the members of the *P. anserina* species complex are shown below as a phylogram [44]. **B** Linear regression showing that TE abundance in the species complex is associated with an increase in genome size (**C**) Linear regression showing that there is no significant association between genome size and *crapaud* abundance

We investigated the TE content in the genomes of the recently diverged *Podospora* species complex [39, 43, 44] (Supplementary Table 1), a survey that up to the point of this study had only been done in *P. anserina* [36]. To assess repeat abundances of all repeat families in the genomes we used the previously published repeat library [39] to mask and retrieve TEs from the genomes. By this analysis, we verified a high and variable abundances of *crapaud* in all genomes of this group, highlighting that this element has been important for the overall repetitive landscape evolution in the *P. anserina* species complex. Specifically, we found that *crapaud* is the repeat family with the largest abundance in terms of base pair coverage in all genomes, except for *P. pseudocomata*, in which *discoglosse*, a DNA transposon, is most abundant (Fig. 1A and Supplementary Table 2). The total base pair coverage of *crapaud* is lowest in *P. pseudocomata* (128,887 bp and 0.37% of total genome size) and highest in *P. pseudopauciseta* (906,421 bp and 2.5% of total genome size) (Fig. 1 and Supplementary Table 2). The total repetitive base pair abundance is low in the *Podospora* genomes (~ 7%) and *crapaud* makes up a large portion of the total repeat abundance: elements of this family comprise between 10 and 58% (average 40%) of the total TE base pair coverage in the seven genomes (Supplementary Tables 1 & 2). The total amount of TEs is also significantly associated with a ~ 5% genome size difference observed in the seven *Podospora* genomes (Linear regression, R^2^ = 0.94, *p* < 0.001) (Fig. 1B). Noteworthy, while genome size correlates with total TE abundance, it is not significantly correlated to *crapaud* (Linear regression, R^2^ = 0.35, *p* = 0.16) (Fig. 1C). The difference in genome size in the species complex is instead significantly associated with the second most abundant repeat, the gypsy/Ty3 LTR-retrotransposon *grenouille* (Supplementary Fig. 1) (Linear regression, R^2^ = 0.91, *p* = 0.0042). The reason for the lack of strong correlation between *crapaud* abundance and genome size in this group could be that *crapaud* proliferation is older and may have spread in the common ancestor of *Podospora*. The *grenouille* element varies more in abundance throughout the species complex than *crapaud*, and is abundant in the three genomes with the largest genome size and less abundant in the other four. These contrasting results indicate that the distribution of *crapaud* most likely reflects high ancestral activity with lower species-specific activity.

### A sequence similarity network (SSN) of the *crapaud* terminal repeats defined 14 distinct, but connected, subfamilies

**Fig. 2:**
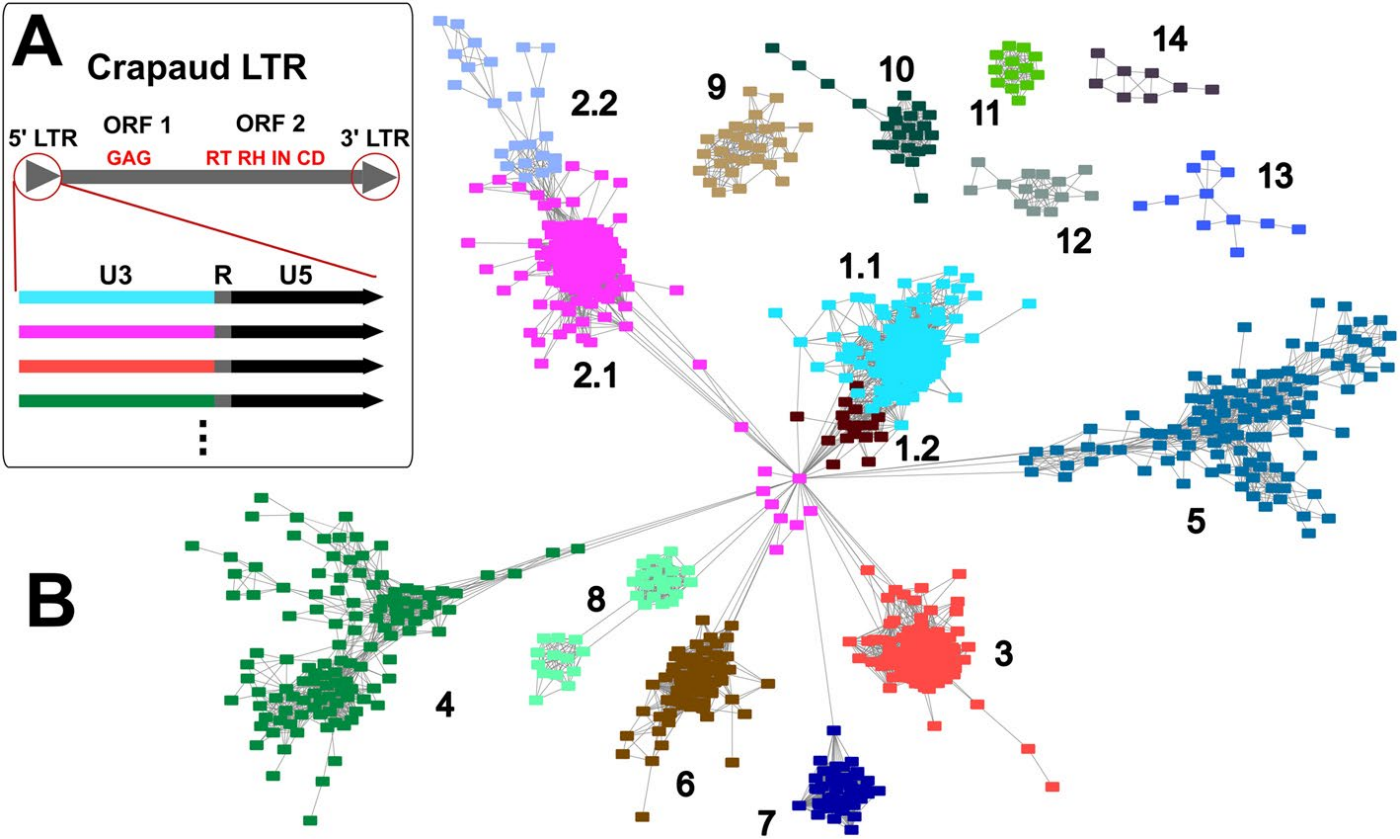
Structure and similarity clustering of *crapaud* LTR copies. **A** A diagram of the *crapaud* LTR-retrotransposon depicting the two ORFs, one coding for a GAG protein and the other for the four domains: Reverse Transcriptase (RT), RNAse (RN), Integrase (IN), and a Chromodomain (CD). The inset shows the LTRs with subfamilies illustrated in different colors. **B** Sequence similarity network of *crapaud* terminal repeats. Sequences (nodes) with > 80% percent identity over > 80% of sequence coverage are connected together with edges. Colors and community numbers are determined by an unweighted greedy modularity community algorithm [46, 47]

As previously reported, we found that the variation of *crapaud* in *P. anserina* is located primarily in the terminal repeats of the element [39], and after a closer inspection of *crapaud* from all investigated genomes in this study, we found that the variation is mostly limited to the U3 region (5’-half) of the terminal repeat, preceding the TATA-box (Fig. 2A, Supplementary Fig. 2). By using a specific BLAST approach of the conserved 3’-half of the element to identify *crapaud* terminal repeat sequences in each of the seven genomes, we retrieved 1455 terminal repeat copies after our manual curation (see Methods). In a second round of copy retrieval, we used the same approach to perform a BLASTn search of the internal region of the originally classified *crapaud* sequence to determine if the 1455 terminal repeats were part of a full copy or if they were solo/fragment LTR copies. By performing this second round we retrieved 150 full copies (i.e., harboring two flanks and an internal region) of *crapaud* from the seven *Podospora* genomes. As during LTR-retrotransposition the 5’-LTR of the newly formed copy is synthesized from the 3’-LTR template [45], we expected the two LTR copies to be identical after insertion, and hence we only kept 5'-LTR [45] per full copy for the terminal repeat classification. Thus, in the final *crapaud* terminal repeat dataset, including the 5’-terminal repeat of full copies and all solo/fragment copies from all seven species, a total of 1305 terminal repeat sequences were used for a more systematic classification of the *crapaud* terminal repeats and for subsequent analysis of their evolution (Supplementary Table 3). Note that while we did not include both terminal repeats of full copies, we investigated them further and surprisingly found seven copies out of the 150 full copies for which the 5’ and 3’-ends are disparate. Based on the identical LTRs left by the transposition mechanism, we expect that this LTR pattern is the result of gene conversion or ectopic recombination.

Illustrating the difficulty of classifying the *crapaud* LTR sequences, clustering using recommended sequence clustering [48] detected 292 clusters at 80% similarity and 80% coverage thresholds. By comparison, using a sequence similarity network (SSN), with 80% identity over more than 80% of the length, we identified 16 distinct but interconnected communities of *crapaud*, each containing more than five copies (Fig. 2B). Communities are a property of networks that identifies groups of nodes in a network that are more connected to each other than those outside of the community. It does not necessarily reflect biologically relevant groupings, but are expected to be correlated with such relationships. For example, two pairs of communities were tightly nested in the network compared to the rest: 1.1 and 1.2, and 2.1 and 2.2. To investigate how these nested communities were distinct from each other we aligned the centroid sequences of the communities, which are the sequences with highest numbers of connected edges from its node. With this approach, we verified that communities 1.1 and 1.2 and communities 2.1 and 2.2 are highly similar but with minor structural differences (Supplementary Fig. 3), and accordingly, we merged them into the joint subfamilies LTR1 and LTR2, respectively. Hence, from the 16 communities we ended up with 14 final subfamilies based on the terminal repeat variation (Supplementary Table 2). This is two more than the 12 annotated terminal repeats in the previous repeat library [39], and 11 of them correspond with the previous classification (Supplementary Table 2). To ensure that the copies of some of the smallest subfamilies consisted of unique insertions and not simply the same ancestral insertion present in different genomes, flanks were extended and aligned. Subfamilies 10–14 all had more than five unique insertions and subfamilies 1–9 all had multiple copies in individual species and a high total count (> 24), indicating that these subfamilies are not composed entirely of orthologous sequences. Altogether, after the above classification steps, 1079 of the 1305 *crapaud* terminal repeat sequences were classified while 226 sequences were left unclassified and labeled as “Unclassified *crapaud*”.

Unlike consensus-based classification of TEs, the SSN method evaluates the similarity between all copies. This approach has several advantages: (1) It does not depend on the assumption that the consensus sequence represents the ancestral sequence of a given TE family; (2) It can capture stepwise copy sequence divergences within a TE family, such as the divergences caused by the effects of RIP. Furthermore, it is possible to use network algorithms and network statistics to infer communities and the relationship between copies, and as we have shown can also be used to detect and classify subfamilies. It is important to note, however, that the SSN we have used differs from a phylogenetic network. The SSN does not incorporate models of evolution and only connects sequences by sequence similarity and coverage thresholds. The SSN is thus more susceptible to reticulations due to the highly similar nature of copies, which may or may not be due to recombination and horizontal transfer. SSN is not the single solution to the problem of classification of TEs with complex evolutionary histories. For example, in our results, LTR1 and LTR2 were split in different communities by the network algorithm meaning that multi-sequence alignments were needed to complement the classification process. Another area of development using the SSN approach in the future is how to deal with low frequency indels. If the indel covers enough of the sequence it will be left unclassified by the SSN. However, other methods such as using phylogenetic groupings to classify subfamilies, which also do not require a consensus sequence, similarly runs into the problem of determining where to draw the line between subfamilies. In essence, the SSN is a powerful method to handle divergence of RIPed copies, but it can be developed further. Our results suggest that the combination of the SSN, usual alignment-based methods, and phylogenetics is needed to sort out complex sequence relationships.

### A maximum likelihood phylogeny largely supports the SSN classification and the divergence of *crapaud* into subfamilies

**Fig. 3:**
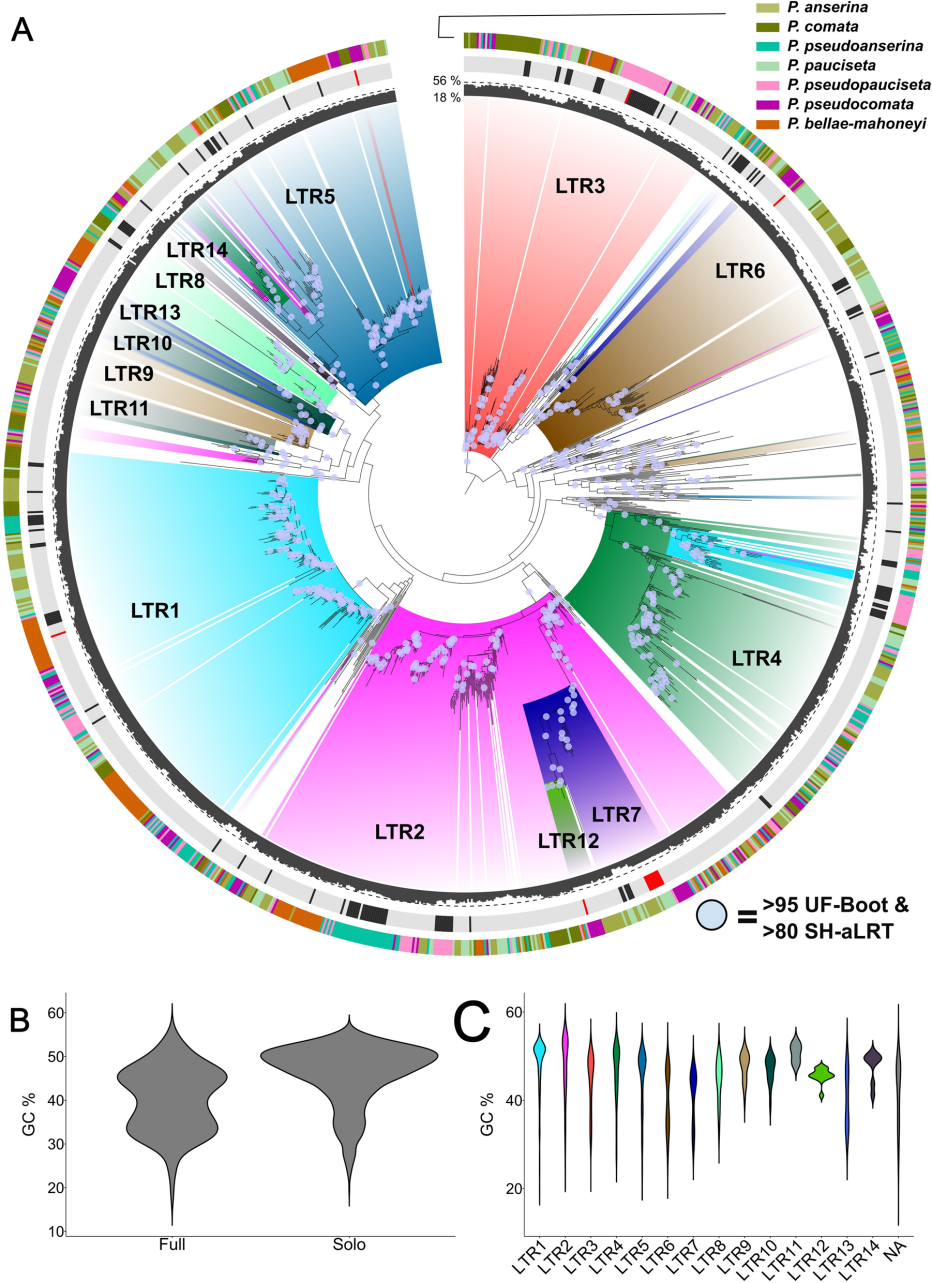
Evolutionary history of the *crapaud* LTR subfamilies (**A**) Maximum likelihood phylogeny of *crapaud* terminal repeats, arbitrarily rooted in the branch leading up to the LTR3 clade. Outer track: Species; middle track: Solo/fragment (grey), full (black), active (red); Inner track: GC-content (%), scaled between lowest (18%) and highest (58%) of the *crapaud* LTRs. Colours in the phylogeny correspond to the LTR subfamilies, and large clusters of a subfamily are annotated with the name of the subfamily. Branches with UFBoot values above 95 and SH-aLRT support above 80 are shown with blue circles. **B** Violin plot showing distribution of GC content in full copies and in solo/fragment copies. **(C)** Distribution of GC-content in the different subfamilies. Unclassified copies are labeled as NA.

We generated a maximum likelihood (ML) phylogeny of the 1305 *crapaud* terminal repeat copies. To identify a sequence for rooting of the LTR phylogeny we performed a BLASTn search against other Sordariomycete genomes in the Mycocosm database [49] using the original *crapaud* terminal repeat as query. The results suggest that members of the different *crapaud* subfamilies are present in other species outside of the *P. anserina* species complex (data not shown), which makes it difficult to select an element representing a sister group to all *crapaud* copies in *Podospora*. We therefore opted to root the tree in one of the subfamilies identified herein, LTR3, where most copies are grouped together with a long supported branch suggesting that the clade is unlikely to be paraphyletic. The resulting ML phylogeny shows that while many of the 226 Unclassified *crapaud* copies cluster outside of the subfamily clades and have long branches, the 1079 classified subfamilies grouped together in monophyletic groups with good bootstrap support, with some exceptions (Fig. 3A). First, some clades of subfamilies are nested in others (e.g., LTR12 is monophyletic but nested in LTR7, and their common clade is nested in LTR2, making LTR2 paraphyletic). Furthermore, in several subfamilies a small set of copies groups outside of their main subfamily clade (these are henceforth called “rogue” copies). For example, LTR1 harbors 27 copies that group together with the subfamily LTR4 instead of with the rest of the 397 LTR1 copies. Likewise, LTR4 has a clade with 12 rogue copies nested in the main LTR5 clade and LTR2 has multiple rogues clustering together with the LTR5 subfamily and others forming a small clade of their own. The only subfamily that did not have the majority of the copies grouped into one clear clade was LTR13. Members of this subfamily are instead scattered within the LTR10 clade and cluster together with some of the long branches leading up to unclassified crapaud copies.

We found that rogue copies have lower degree values in the SSN (i.e., lower number of other sequences they are connected to in the network), as compared to the copies clustering with the main subfamily clades in the phylogeny (Wilcoxon rank sum test, *p* = 2.2 × 10^−16^) (Supplementary Fig. 4B) in agreement with the relatively weak assignment of these into the clusters. In terms of sequence context, the rogue copies have lower GC-content than non-rogue copies (Wilcoxon rank sum test, *p* = 2.2 × 10^−16^) (Supplementary Fig. 4A), suggesting that RIP has an impact on their phylogenetic placement. Furthermore, we made alignments of the rogue copies and the centroid of the original classification of the copy, and compared that to the alignments to the centroid of the subfamily that the rogue copy grouped within the phylogeny. With this analysis, we found that the LTR1 rogue copies grouping with LTR4, and rogue LTR4s grouping with LTR5 are more similar to the centroid of their own subfamily than with the one they share clade with in the phylogeny (Supplementary Fig. 5A-D). However, these rogue copies show structural differences such as indels, fragmentation and duplications compared to the centroid of their own subfamily suggesting that degradation, rearrangements, and nested insertions have an impact on inferring the phylogenetic relationship of the *crapaud* elements. In contrast, the U3 region of the LTR3 rogue copies mostly share similarity with the LTR3 centroid and partly to the LTR5 copy, suggesting that these are recombinants between the two (Supplementary Fig. 5E-F). Finally, we note that the rogue copies of LTR2 identified as outliers in the ML phylogeny, are the same copies as found in the middle of the SSN and connected to multiple subfamilies (Fig. 2B). By aligning these copies to the centroid sequences of all the subfamilies we found that each has unique patterns of fragmentation, duplications and inversion, similar to the other rogue copies. These patterns indicate that the LTR2 copies in the middle of the SSN should not be seen as ancestral copies to multiple subfamilies but rather artifacts created by the aforementioned patterns (Fig. 2B).

In order to evaluate how well our classification based on a curated dataset of copies represents all *crapaud* copies in the genomes, we used the centroids of the subfamilies to do a RepeatMasker search of the genomes [50]. Additionally, the RepeatMasker search allowed us to compare our classification to RepeatMasker’s assignment of the same copies. In total, RepeatMasker identified 2402 terminal repeat copies, which is 1582 copies more than what was used for the classification (Supplementary Fig. 6). These include both the copies used in the curated dataset and those too fragmented for our curation criteria. The overall proportion in abundances of subfamilies were consistent with what is seen in the curated dataset. RepeatMasker classified 62 out of the 1079 classified copies differently from the SSN, representing an error rate of 5.75% (Supplementary Table 3). This low but noticeable level of disparity between our classification and RepeatMasker masking highlights that masking RIPped sequences can give erroneous classification. These errors are also likely to be more numerous if there is complex variation and other differences between copies such as fragmentation, rearrangements, and nesting. Particularly with regards to RIP, the scoring matrices used in RepeatMasker are based on a set background GC-content level [50]. There is an option in RepeatMasker to calculate and use the average GC-content of a batch of sequences, although this requires further testing.

In summary, our data suggest that there are three main reasons as to why there are contrasting patterns between the SSN, the ML analyses, and the RepeatMasker output: (1) mutations caused by RIP, (2) indels and other rearrangements, (3) recombination between elements of different subfamilies, and (4) methodological differences in scoring relationships between copies. It has previously been argued that monophyly should define TE classification [28]. In the presence of RIP, the SSN analysis offers an additional approach to classification, which has allowed us to identify a higher number of copies in the assemblies. Our results thus suggest that the SSN is a valuable addition to other methods, such as multiple sequence alignments, to efficiently classify complex LTR-elements in fungi. The ML phylogeny was also an important verification to show that the SSN clusters had phylogenetic meaning.

### Analyses of *crapaud* sequences give insight into the mechanism and process of diversification of LTR-elements

The expectation that both terminal repeats are identical upon insertion has previously formed the basis of estimating the age of LTR copy insertions by comparing their divergence [51–54], and relies on the assumption that the sequences evolve neutrally. From the SSN, the ML phylogeny and the alignments, we see that the LTRs of *crapaud* have diversified into multiple subfamilies with differences concentrated on the U3 region, which suggest that there is a complex selective landscape across the LTR, and that the terminal repeats are not necessarily evolving neutrally. In addition, as mentioned above, we found full copies with the two terminal repeats assigned to different subfamilies, suggesting that gene conversion of the terminal repeats has occurred between subfamilies. In a previous study of *Saccharomyces cerevisiae*, it was found that there is a negative relationship between copy length and LTR similarity, and the authors argued that gene conversion can disrupt the assumptions made for age estimations in copies of LTR retrotransposons [54]. The patterns we observe could indicate both diversifying selection on the U3 section of the LTRs and gene-conversion between copies, and further support the notion that the assumption for LTR age estimations based on the idea of a molecular clock should be carefully considered. Additionally, the finding of recombinant terminal repeat copies of different subfamilies may provide insights into how *crapaud* diverged into subfamilies. During reverse transcription of LTR-retrotransposons the 3’-half of the LTRs are reverse transcribed first before a template switch to the 5’-half. During this template switch, it has been suggested that there could be an opportunity for recombination between LTRs if two heterologous RNA molecules of the LTR-retrotransposon were packaged together within the same virus-like GAG particle [55]. In the Ty1 element in *S. cerevisiae*, promoters make up a region spanning both upstream and downstream of the transcription start site, with some transcription factors binding partly or exclusively in the U3 region of the LTRs [56]. A hypothesis for the evolution of the diversified region in *crapaud* is that it has evolved through selection for a more efficient or specific promoter or enhancer function. For example, the *S. cerevisiae* Ty1/Ty2 LTR-hybrids have more transcription factor binding sites than the non-hybrid copies, which is reflected as a higher expression rate than either of the parent elements Ty1 and Ty2 [56, 57]. The details of promoter binding sites and expression rates of different subfamilies of *crapaud* is unknown, but one can imagine a scenario where competition between elements or evolution for different expression patterns has shaped the evolution into subfamilies and the burst patterns we observe. Another possibility is that the subfamilies evolved by chance as a result of a historical period of high activity of *crapaud* where multiple recombination events of copies happened in a short time frame and then each of those recombinants later continued transposing with strong purifying selection and/or gene conversion keeping the R and U5 regions conserved among the different subfamilies. Additional analyses of transposition and evolutionary trajectories of *crapaud* elements would be of interest and can help disentangle the different evolutionary forces shaping LTR-elements in general, and the *crapaud* elements of *Podospora* in particular.

### The *crapaud* subfamilies diverged prior to the diversification of the *P. anserina *species complex and have had consistent burst activity during the evolution of the species complex

As mentioned above, the presence of *crapaud* subfamilies outside of the *P. anserina* species complex suggest that the diversification into the different subfamilies happened before the diversification of the species. This notion is strengthened by the lack of a strong correlation between the abundance and genome-size of the abundant element *crapaud* in the *P. anserina* species complex, which suggests historic proliferation. The patterns observed in the ML phylogeny can further help in studying the timing of diversification and the activity of *crapaud* in relation to the time of species diversification. Firstly, most subfamilies are present in all seven species and their diversification pattern follows the expected species relationship suggesting that these copies had diverged into subfamilies prior to diversification of the *P. anserina* species complex. An example of this pattern is LTR1, which has copies present in genomes of all seven species. Furthermore, the two subclades of LTR1 are also present in all *Podospora* genomes, which suggests that the division into these subclades is also ancestral to the species complex (Fig. 3A, supplementary Fig. 7). Another clade showing ancestral proliferation is LTR2-LTR7-LTR12 clade where there is a lack of species-specific clustering close to the root of the clade (Fig. 3A, Supplementary Fig. 8), strongly suggesting that this group of elements was also present in the ancestor of the species complex. Noteworthy, both these example clades also show copies from sister species clustering together (Fig. 1A), which suggest proliferations of the ancestral variants over the same evolutionary time as diversification of the species complex. Specifically, the sister species *P. anserina* and *P. pauciseta* share a burst in subclade 2 of LTR1. Likewise, the three species *P. pseudoanserina*, *P. pseudopauciseta* and *P. pseudocomata*, which form a group in the species phylogeny (Fig. 1A), are closely grouped together in both subclades of LTR1 (Supplementary Fig. 7), as well as in LTR2 (Supplementary Fig. 3). These patterns are indicative of bursts taking place after the early diversification of the species complex, but before the split of the most recently diverged species.

At the species level, there are also multiple examples of individual bursts of subfamilies, some of which can be interpreted as more recent than others (Fig. 3A and Supplementary Figs. 7, 8). For example, copies of LTR1 in *P. bellae-mahoneyi* have two different burst patterns (Fig. 3A and Supplementary Fig. 7). The first one, in subclade 1, is composed solely of solo/fragment copies, i.e., likely showing a burst where all copies have undergone LTR-LTR recombination or are fragmented. This burst only contains solo/fragment copies suggesting that it is an older burst or that selection has been more effective at removing the full copies than during other bursts. Similar expansions are also present throughout the phylogeny of many of the subfamilies including several of the smaller subfamilies that lack full copies altogether. For example, the second burst of LTR1 in *P. bellae-mahoneyi*, has multiple solo/fragment copies, multiple full copies, and even one potentially active copy, determined by the presence of ORFs and protein domains (Fig. 3A and Supplementary Fig. 7). This pattern indicates that this is likely a more recent, and potentially still ongoing, burst of this subfamily in *P. bellae-mahoneyi*. The most striking example of a burst with active copies is in LTR7 (Fig. 3A and Supplementary Fig. 8). The burst in LTR7 is the only one that has more than one potentially active copy, and the recency of this burst is highlighted by the extremely short branches in the clade. The exact timing of the different bursts is unknown but it is clear that they have happened at different points in time relative to the split of the species in the complex. In this burst, we also found putative active copies that are nearly identical between *P. anserina* and *P. pauciseta* (Fig. 3A, Supplementary Fig. 8). It has previously been suggested that these two species have had introgression between them [58], and the identical copies of LTR7 support that there is occasional introgression between these two species further shaping the diversification of *crapaud*.

All genomes in our dataset, except for those of *P. comata* and *P. pseudoanserina*, have at least one copy that could be potentially active. This could mean that *crapaud* is dead in these two species, but it is difficult to conclude since other strains of those species may still carry active copies. For example, in *P. comata* there are several genomic datasets published (although mostly of lower quality), not included in this study, that could be further investigated [39, 43]. The observed species-specific burst patterns also indicate that several of the subfamilies have been consistently active since the split of the species complex. One species, *P. pseudocomata*, has several interesting patterns of dynamics of *crapaud* elements. While it is the species with the least abundance in terms of base pair coverage it has several active subfamilies not active elsewhere, such as LTR6, LTR12, and the copy that is annotated as LTR3 in our dataset but that we showed to be a new recombinant between LTR3 and LTR5. These active copies of *P. pseudocomata* may represent a new cradle for the *crapaud* family to further diversify.

Overall, the patterns of little species-clustering close to the early diverging branches, shared bursts between species consistent with the species phylogeny, and species-specific bursts of individual subfamilies suggest that the *crapaud* subfamilies diverged in the ancestor of the species complex. Based on the abundance of the *crapaud* family throughout the genomes of the species complex and its pre-species complex divergence, we conclude that this TE has been successfully spreading and evolving for a prolonged time, with potential introgression between species. Furthermore, some subfamilies are still potentially active in one or multiple species, indicating that this persistence remains in the face of RIP.

### Contrasting evolutionary patterns of proliferation between *crapaud* and *grenouille* in the face of RIP

The evolutionary trajectory of *crapaud* differs from the *grenouille* LTR element. As mentioned above, *grenouille* shows a strong correlation with genome size (Supplementary Fig. 1), and likely represents a more recently diverged LTR family than *crapaud*. In contrast to *crapaud*, *grenouille* lacks the terminal repeat diversification. Instead, 692 copies out of 753 terminal repeats clustered into one large SSN cluster using the same thresholds (80% identity and 80% coverage) (Supplementary Fig. 9A). In *grenouille*, the second largest SSN cluster consisted of seven copies, each from one of the genomes of the seven species and from the same position in the chromosome, indicative of an orthologous insertion (Supplementary Fig. 9A). The ML phylogeny of *grenouille* copies have large expansions in accordance with the species relationships (Supplementary Fig. 4B).

While the deep relationships between the *crapaud* subfamilies are unresolved, there seems to be two patterns of divergence: one is the ladder-like evolution of the LTR2-LTR7-LTR12 clade with subfamilies evolving from other subfamilies, and the other is star-like, where subfamilies have a single origin branch not stemming from another subfamily. The ladder-like divergence into subfamilies to escape genome defense has been observed and highlighted in previous studies, such as the evolution of mammal L1 non-LTR retrotransposons that have ladder-like, subfamily evolutionary patterns [30, 59, 60]. This ladder-like pattern has been suggested as the result of a host-parasite arms-race to escape genome defense [30, 32, 59, 60]. The *crapaud* element however, show burst-like origins in many of the subfamilies, which suggests that, in contrast to our original hypothesis, it has not evolved due to a host-parasite arms-race to escape RIP. In comparison, the evolutionary trajectory of *grenouille* conforms better to the typical TE that has been suppressed by RIP and may have escaped in only one or two species, with only very few full copies after massive expansions. The *grenouille* repeat has had recent expansions in mainly three of the genomes (*P. anserina*, *P. pauciseta*, and *P. pseudopauciseta*), but not the same continuous activity relative to the species complex diversification as *crapaud* (Supplementary Fig. 4B). There is only one putatively active copy of *grenouille* in *P. pauciseta* in our dataset and almost all other copies have been fragmented or subjected to solo-LTR formation. Thus, the *grenouille* repeat copies have likely been highly targeted by RIP and as a result only one or a few copies have escaped, but not by forming new subfamilies. The persistent activity of the *crapaud* element as indicated by the many expansions in different species hint at a different evolutionary trajectory and that RIP is not the main driver of the *crapaud* subfamilies diversification, although it does affect its proliferation (see below).

### RIP targets LTRs of full copies of *crapaud* but not solo/fragment LTRs

We found that the GC-content of full copy terminal repeats of *crapaud* show a distinct bimodal distribution with few copies with intermediate GC (Fig. 3B). Our phylogeny also shows bursts of elements with varying levels of GC-content, best exemplified in LTR3 in *P. pseudopauciseta* (Fig. 3A and Supplementary Fig. 10). In contrast, solo/fragment copies have mostly copies with high GC and only a minor second peak with lower GC (Fig. 3B). This data indicates that most solo/fragment copies have few or no RIP mutations, likely due to their small size not being recognized by the RIP machinery [19]. Because the divergent U3 region is half the size of the LTR (< 200 bp), it is thus unlikely that the divergence of the U3 was a response to escape RIP but is instead likely driven by other processes. The GC-content in solo/fragment copies of *grenouille* also reflect this pattern (Supplementary Fig. 11). Next we investigated the GC-distribution of terminal repeats of different subfamilies and found that there is a difference in GC-distribution between the subfamilies (Fig. 3C), implying different degrees of RIP targeting.

The bimodal GC distribution in the full copies suggests that some but not all copies have managed to escape RIP and form new expansions after initial RIP mutations. RIP has three inherent features that can allow TEs to escape it. The first is that RIP only acts during the pre-meiotic stage [17], meaning that TEs active during periods of vegetative growth can proliferate without being suppressed by RIP. However, at least *P. anserina* is a species that is obligately sexual and resides in an ephemeral habitat, meaning that there is a limited time period for asexual proliferation during mycelial growth in this species [61]. The second is that RIP has specific requirements for size, similarity, and distance between duplicated copies, meaning that there is a possibility for copies to escape by not being targeted if they are either too small or too dissimilar [20, 24]. Third is that RIP may drive sequences to be divergent enough from each other to avoid being further targeted but still be functional. If the copy still has the machinery necessary for transposition, it can copy itself and form a new burst, and in this way escape from RIP. However, this would produce a ladder-like pattern in the phylogeny of the copies, which is not what we observe.

Another, related, question left unanswered by our result is why there are fewer full copies with intermediate GC-contents than copies with either high or low GC-content. Since RIP is stochastic it would be reasonable to expect a continuous range of GC-content in copies. It has previously been shown that copies with higher AT content trigger a nearly fivefold lower RIP response than those with lower AT [62]. Thus, one explanation could be that RIP has periods of high activity, meaning that there is a large excess of copies not yet targeted by RIP and of copies that have reached an AT-content high enough to not be recognized anymore. Notably, there is a much higher proportion of non-RIPped copies in the solo/fragment copies. If the solo-LTR formation happens only occasionally as a result of intra-copy recombination it should happen for both RIPped and non-RIPped copies. One possibility is that LTR-LTR-recombination resulting in solo-LTRs happens at a much higher frequency than RIP and that in a given burst a majority of the copies become solo-LTRs before being exposed to RIP. A second option is that LTR-LTR-recombination occurs for non-RIPped and RIPped copies alike and that the solo-LTRs back-mutates through gene-conversion over time to have a more uniform GC-distribution. Gene conversion in full copy LTRs have been observed in *S. cerevisiae* to be negatively correlated to the LTR-LTR recombination that forms solo-LTRs [54].

### The evolution of the internal region of the crapaud elements differs from that of their own LTRs

**Fig. 4:**
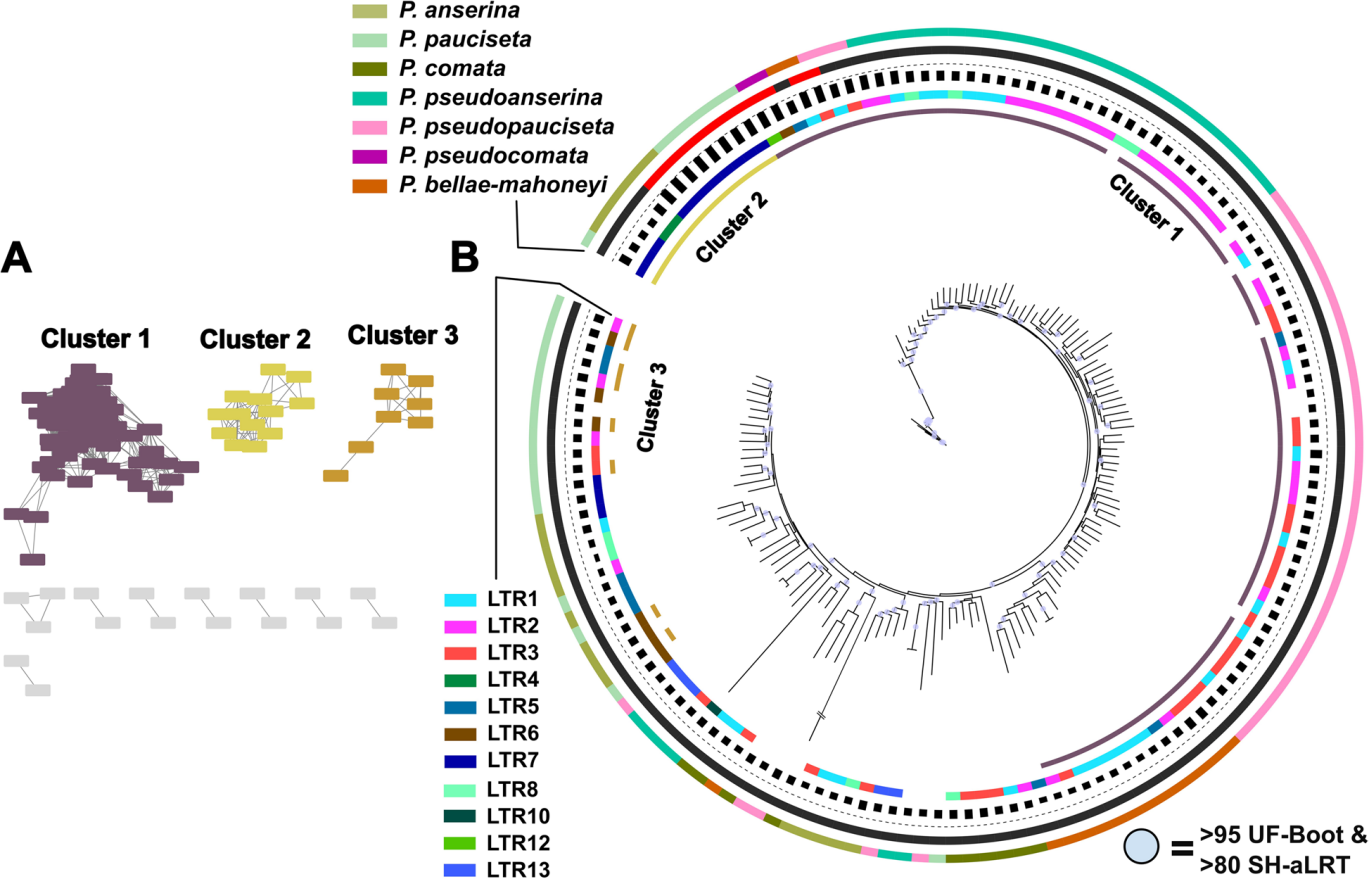
Comparison of *crapaud* internal region of 150 full copies (**A**) SSN clustering based on 80% identity and 70% coverage thresholds. Three clusters with > 5 copies are shown in color, clusters with 2 or more copies are shown in grey. **B** Maximum likelihood phylogeny of the internal region. Tracks from inner to outer: Internal region clusters; LTR subfamily; GC-content, scale ranging between 14 − 54%; Full copies (black) and putative active copies (red); Species

Initial inspection of the full copies of *crapaud* identified further structural differences within the internal region of the element. We found that the internal region of the 150 full copies divides into three main network clusters based on sequence similarity (Fig. 4A). Ninety six of the *crapaud* full copy sequences cluster into the largest network cluster; the second and third largest clusters contain 19 and 13 sequences, respectively. Cluster 1 contains sequences from all seven species, while cluster 2 and 3 contain sequences from only *P. anserina* and *P. pauciseta*. Alignments of the internal regions revealed that Cluster 1 and 2 align well except for a ~ 1.1 kb deletion in sequences of cluster 2. Cluster 3 aligns poorly with the other two clusters in several regions of the alignment. We find that these clusters were also supported by a maximum likelihood phylogeny where Cluster 2 is nested within Cluster 1 (Fig. 4B). Many of the sequences did not cluster in the SSN, likely an effect of being heavily RIPped and fragmented. Despite this, sequences in the ML phylogeny are grouped based on species rather than LTR subfamilies within internal region clusters. This suggests that the relationships we reported from the LTR subfamilies do not extend into the rest of the full elements. Notably, all the putatively active copies cluster closely together in the phylogeny.

To explain the disparity between the evolutionary trajectories of the LTR and the internal region of *crapaud*, it is important to understand the differences between the two regions of the TE: (1) RIP will have different profiles between full copies and solo/fragment copies, where RIP is more prevalent in the full copies. This means that in our LTR datasets, where solo/fragment copies are overrepresented, RIP will have a smaller effect on estimating evolutionary relationships in the maximum-likelihood phylogeny. (2) The majority of the internal region is homologous between the subfamilies (with the exception of the structural differences described above) and there is little association between the relationships of the terminal repeats and the internal regions of copies. This indicates that the terminal repeats and internal region of the element are likely under different evolutionary constraints. There may be some homogenizing effect on the internal region of the element through gene conversion between copies of different subfamilies in a given species. RIP may also not discriminate between the full copies carrying LTRs of different subfamilies when inducing mutations. Since the terminal repeats together only account for 800 bp (~ 11%) of the total full copy sequence length, copies of different subfamilies could still share enough similarity to be recognized by RIP. If one subfamily has an expansion, copies of all subfamilies in the species may be exposed to similar levels of RIP, which would also contribute to having a homogenizing effect. (3) Methodologically, the SSN has trouble capturing the relationship of the internal region due to more low-frequency indels. A better handling of indels would give a more accurate view on the relationship in the internal region between the copies and their evolutionary relationships.

## Conclusion

In this study we have comprehensively classified the *crapaud* LTR family, which has a complex evolutionary history that has led to the evolution of subfamilies defined by different terminal repeats. The presence of both the different terminal repeats and the high mutation rates caused by RIP demanded additional methods apart from the usual consensus-based approach. Here we successfully developed an SSN approach that we used together with more typical classification methods. Our findings show that *crapaud* has had both ancestral diversification and recent, species-specific bursts, some of which are still potentially ongoing. The short divergence time of the *P. anserina* species complex, as indicated by their high synteny and low sequence divergence (> 98% in genic regions) [43], makes it an ideal system to study the short-scale perspective needed to understand fast evolving TEs. In the last few years, there have been several similar studies that investigate the short-scale evolutionary dynamics of LTR-retrotransposons [63, 64]. This phenomenon of LTR variability has been reported in at least one other species [65], thus these patterns may be widespread and should be investigated further. A similar process of LTR-hybridization through inter-LTR recombination was observed first in yeast [55], opening up the question whether this phenomenon is more widespread in other organisms. The diversification of *crapaud* into subfamilies based on differences in the terminal repeats currently presents a unique opportunity to learn about other aspects of LTR-retrotransposon evolution, and how subfamilies evolve.

## Methods

### LTR abundance analysis

To describe the levels of LTR abundances, we used the previous repeat library (PodoTE v1.0) [39]. In the repeat library the terminal repeats of LTR retrotransposons were annotated separately from the internal region of the element for masking purposes. For the *crapaud* element the terminal repeat variations were annotated separately using the names: [Tamasoli_LTR, Sapo_LTR, Rupikonna_LTR, Mainduk_LTR, Kaeru_LTR, Padda_LTR, Dukkeobi_LTR, Crapaud_LTR, Hama_LTR, Hikigaeru_LTR, Tudse_LTR, Krote_LTR]. RepeatMasker v4.1.4 [50] was used to calculate abundances of TE-families in the *P. anserina* species complex genomes [39, 43, 44], with default parameters followed by filtering out simple repeats. For LTR-retrotransposons, we combined the separate annotations of the internal regions and the terminal repeats to get overall abundances of those repeat families. The abundances were visualized using the ggplot2 v3.4.2, ggpubr v0.6.0, and ggrepel v0.9.3 in R v4.3.0. The same steps were also implemented for the *grenouille* repeat element.

### Retrieval of *crapaud* LTR and full copies from seven *Podospora* genomes

To retrieve both LTR sequences and full copy sequences, the originally annotated *crapaud* sequence [36] (Genbank accession: EU697463.1) was used as a query for retrieval of both LTR sequences and full copy sequences. From the repeat library, the terminal repeats annotated were collected and aligned. The conserved 3’-half of the *crapaud* terminal repeat was then used as a query for retrieval of terminal repeats. Copies were retrieved by using the custom script, query2haplotype.py v1.41 (https://github.com/SLAment/Genomics) with the -H option to retrieve haplotypes and otherwise default settings. The script utilizes BLASTn v2.5.0 [66], then merges together the hits into haplotypes and then finally extends the hits. The retrieved copies then went through a first curation step using MAFFT v7.310 [67] to reverse complement sequences to the direction of the query sequence and then by using the dotplots generated in the online MAFFT web application v7 [68] split copies sitting close together that were merged together by the script. To accurately find the start and end of the terminal repeat sequences, several rounds of manual alignment using the mafft-add function [69] were done. For each step of this process, sequences were aligned and those sequence ends of the terminal repeat that aligned to at least one other sequence and could accurately be determined were cut out and aligned separately, then the rest of the sequences were added to the cut out alignment using MAFFT-add. In the end we kept sequences that aligned to at least one other copy in the U3 region of the terminal repeat and where we could find the terminal repeat end, as characterized by target site duplications and/or the TpG motif typical at the start of LTR-retrotransposon copies, while also being mindful of RIP turning the G in the TpG motif to an A in some copies. The query2haplotype.py script also has the feature that the sequences it extracts have the name structure: “contig_start-end”. BLASTn self-hits were collected for all sequences after manual curation and then used to update the start and end positions after manual curation.

To annotate terminal repeats as full copies of solo/fragment copies a second round of copy retrieval was done. By instead using the internal region of the *crapaud* repeat as query in the query2haplotype script with a minimum size (-s) of 4 kb and extending the haplotypes by 2 kb (-f). The retrieved haplotypes went through a similar manual curation as for the terminal repeats, by (1) aligning the haplotype sequences using MAFFT and removing hits that were too fragmented and/or nested, and (2) Removing sequences that missed one or both terminal repeats.

### Network construction and subfamily classification

To classify the curated sequences into subfamilies, a sequence similarity network was built. This was done in three steps following a modified pipeline from https://github.com/MiguelMSandin/SSNetworks. This pipeline includes three main scripts for building networks: 1.1_blastn_allAgainstAll.sh, 1.2_blastClean.py and 2.1_buildNetwork.py. The first script uses an all-vs-all BLASTn homology search to get the level percent identity between each sequence, the search algorithm of BLASTn was modified to blastn instead of Megablast in the original pipeline as it is preferred for TE sequences. The second script cleans the output by removing self hits and reciprocal hits and was modified to handle fragmented and overlapping hits between sequences that are prevalent due to RIP and the fast evolution of TEs. The third script builds the final network by connecting sequences by both coverage and percent identity thresholds. This script was modified to recalculate percent identity score based on coverage and percent identity of all fragmented and overlapping hits (excluding overlaps) and not only to the top hit.

For the *crapaud* terminal repeat SSN, thresholds of > 80% identity and > 80% coverage were used. For the internal region network, percent identity > 80% and coverage > 70% were used. In addition, due to the interconnectedness of the terminal repeat network a greedy modularity community (GMC) [46, 47] algorithm in the NetworkX v2.6.3 [70] python package was implemented to find communities within the resulting network. The greedy modularity community detection algorithm starts with single nodes in the network and then merges nodes along their edges based on an increasing modularity score until the modularity score can not be increased anymore. Community clusters with less than five sequences were left as unclassified *crapaud* sequences in subsequent analyses. Further analysis of the networks and communities within the networks was done in the software Cytoscape v3.9.1 [71], which was also used to analyze network statistics. In the visualization of the SSN the Prefuse Force Directed Layout, which is the default in Cytoscape, was used. To compare the SSN to other sequence clustering, we used the tool CD-HIT-EST [72] with 80% identity and 80% coverage thresholds, as recommended by Goubert et al. [48].

### Phylogeny construction of terminal repeats and internal region

A solo/fragment copy maximum likelihood phylogeny was constructed using IQtree v2.2.0.4 [73] with the parameters: -m GTR + F + R8 -alrt 1000 -B 1000 -nm 30,000 -nt 10. Both ultrafast bootstraps [74] and an SH-like approximate likelihood ratio test [75] were used (the -B and -alrt options) to evaluate branch supports. For the phylogeny of the internal region of *crapaud* (Excluding the LTRs), we used the web application of IQtree v1.6.12 [76] using the model finder option, number of bootstrap replicates of 100, number of bootstrap iterations at 5000, and number of bootstrap replicates for the SH-like approximate likelihood ratio test at 1000. The substitution model found to be the best by the model test was the GTR + F + I + G4 model.

### Repeatmasker estimation of copy number variation between species and subfamilies

To more accurately estimate copy number and abundance of different subfamilies in the seven species, RepeatMasker v4.1.4 [50] was used with a custom library consisting of the subfamily sequences with the highest degree, i.e. the most connections, for each subfamily respectively.

A custom script was made to process the RepeatMasker output in three ways before analyzing it: (1) Since the length of the LTRs of the subfamilies ranged between 339 and 443 bp, hits were filtered to sequences > 250 bp to only count hits that extended into the variable U3 region of the LTR; (2) Matching the hit to the positions of annotated full copies to either count the hit as a full copy LTR if they overlap with the ends of the full copy or solo/fragment if there is no overlap or overlap with the internal region, i.e., a nested copy; (3) Further filtering was done to remove potential solo-LTRs overlapping with the start and end positions of the full copies by comparing with the known terminal repeat classification from the previous analyses; (4) Finally, an output file was created containing counts of the subfamilies in all the seven species of the species complex.

### Supplementary Information

**Additional file 1: Supplementary table 1.** Information on the seven high quality genomes of the Podospora anserina species complex. **Supplementary Table 2.** Transposable element abundances in the Podospora anserina species-complex based on the RepeatMasker [49] output of the Podospora repeat library [41]. **Supplementary table 3.** Information on the sequences classified by the sequence similarity network with communitiesdetected by  the GMC algorithm in the python package NetworkX [68-70]. Shown are the number of full and active copies in each community, the final classifiaction names used in this study and in the updated repeat library, and the best hit to the old library LTR sequences. **Supplementary table 4.** Misclassifications between SSN and RepeatMasker [49] and the number of copies of respective misclassification.

**Additional file 2: Supplementary figure 1.** The LTR element grenouille abundance in base pairs is significantly associated with genome size in the species complex. Pearson´s correlation, p= 0.0042, *R* = 0.91. **Supplementary figure 2.** Alignment of Crapaud subfamily centroids. Top = 5’-half, bottom = 3’-Half. The different colors represent the nucleotides; C = blue, G = black, T = red, and A = green. The TATA-box is marked with a red arrow. **Supplementary figure 3. **Dotplots between the nested cluster centroids. Red lines indicate alignments between the sequences in the forward strand. Blue lines indicate alignments in the reverse strand **A) **Community 1.1 and Community 1.2. **B) **Community 2.1 and Community 2.2. **Supplementary figure 4.** Comparison between classified copies inside and outside their subfamily’s main clade in the ML phylogeny. **A)**GC content comparison, mean (Non-Rogue) = 48.4, mean (Rogue) = 37.4. **B) **Number of edges connected to the sequence in the SSN (Degrees), median (Non-Rogue) = 36, median (Rogue) = 6. n(Non-Rogue) = 997, n(Rogue) = 82. **Supplementary figure 5. **MAFFT alignment dotplots between representative rogue copies, the subfamily centroid, and the centroid of the clade it clusters with in the ML phylogeny. **A) **LTR1 rogue with LTR1 centroid. **B) **LTR1 rogue with LTR4 centroid. **C) **LTR4 rogue with.LTR4 centroid. **D) **LTR4 rogue with LTR5 centroid. **E) **LTR3 rogue with LTR3 centroid. **F) **LTR3 rogue with LTR5 centroid. **Supplementary figure 6.** Number of Repeatmasker hits of LTR subfamilies in the *P. anserina *species-complex of both full copy LTRs and solo/fragment LTRs. **Supplementary figure 7. **Pruned phylogeny of the LTR1 clade. Tracks from inner to outer: GC-content, Solo/fragment (grey) / Full (black) / Active (red), Species. Species are also indicated by colors of branches. The phylogeny was rooted based on the phylogeny including all LTR copies of the dataset. **Supplementary figure 8. **Pruned phylogeny of the LTR2, LTR7 and LTR12 clade. Tracks from inner to outer: GC-content, Solo/fragment (grey) / Full (black) / Active (red), Species. Species are also indicated by colors of branches. The phylogeny was rooted based on the phylogeny including all LTR copies of the dataset. **Supplementary figure 9. **Analysis of the *grenouille *LTR element **A) **SSN of the *grenouille *terminal repeats using the thresholds >80% identity over >80% sequence coverage. 692 terminal repeat copies clustered in the largest network cluster. The second largest had seven copies. *n*= 753. **B) **ML phylogeny of the 753 terminal repeats of grenouille. Tracks from inner to outer: GC-content ranging between 18% to 53%; Full copies (black), putative active copies (red), and solo/fragment copies (grey); Species. **Supplementary figure 10. **Pruned phylogeny of the LTR3 clade. Tracks from inner to outer: GC-content, Solo/fragment (grey) / Full (black) / Active (red), Species. Species are also indicated by colors of branches. The phylogeny was rooted based on the phylogeny including all LTR copies of the dataset. **Supplementary figure 11. **GC% content of the terminal repeat sequences of the *grenouille*element. divided into solo/fragment copies and full copies. n(solo/fragment) = 736, n(full) = 17.

## Data Availability

Repository links of the *Podospora* genomes can be found in Supplementary Table 1. The scripts used during this study can be accessed at: https://github.com/Ivwster/Crapaud_TEs. Supplementary data folder with generated data submitted with the manuscript, all relevant files have been included.

## Abbreviations

TE: Transposable element
MSUD: Meiotic silencing by unpaired DNA
RIP: Repeat induced point mutations
MIP: Methylation induced premeiotically
ORF: Open reading frame
SSN: Sequence similarity network
LTR: Long terminal repeat
ML: Maximum likelihood
GMC: Greedy modularity community
