# Supplementary material for: Evolutionary dynamics of the LTR-retrotransposon crapaud in the Podospora anserina species complex and the interaction with repeat-induced point mutations
Source: Mob DNA. 2024 Jan 13;15:1. doi: 10.1186/s13100-023-00311-8 (PMC10787394; doi:10.1186/s13100-023-00311-8)
Supplement: Supplementary file 2 — Additional file 2: Supplementary figure 1. The LTR element grenouille abundance in base pairs is significantly associated with genome size in the species complex. Pearson´s correlation, p= 0.0042, R = 0.91. Supplementary figure 2. Alignment of Crapaud subfamily centroids. Top = 5’-half, bottom = 3’-Half. The different colors represent the nucleotides; C = blue, G = black, T = red, and A = green. The TATA-box is marked with a red arrow. Supplementary figure 3. Dotplots between the nested cluster centroids. Red lines indicate alignments between the sequences in the forward strand. Blue lines indicate alignments in the reverse strand A) Community 1.1 and Community 1.2. B) Community 2.1 and Community 2.2. Supplementary figure 4. Comparison between classified copies inside and outside their subfamily’s main clade in the ML phylogeny. A)GC content comparison, mean (Non-Rogue) = 48.4, mean (Rogue) = 37.4. B) Number of edges connected to the sequence in the SSN (Degrees), median (Non-Rogue) = 36, median (Rogue) = 6. n(Non-Rogue) = 997, n(Rogue) = 82. Supplementary figure 5. MAFFT alignment dotplots between representative rogue copies, the subfamily centroid, and the centroid of the clade it clusters with in the ML phylogeny. A) LTR1 rogue with LTR1 centroid. B) LTR1 rogue with LTR4 centroid. C) LTR4 rogue with.LTR4 centroid. D) LTR4 rogue with LTR5 centroid. E) LTR3 rogue with LTR3 centroid. F) LTR3 rogue with LTR5 centroid. Supplementary figure 6. Number of Repeatmasker hits of LTR subfamilies in the P. anserina species-complex of both full copy LTRs and solo/fragment LTRs. Supplementary figure 7. Pruned phylogeny of the LTR1 clade. Tracks from inner to outer: GC-content, Solo/fragment (grey) / Full (black) / Active (red), Species. Species are also indicated by colors of branches. The phylogeny was rooted based on the phylogeny including all LTR copies of the dataset. Supplementary figure 8. Pruned phylogeny of the LTR2, LTR7 and LTR12 clade. Tracks from inner to outer: GC [file 13100_2023_311_MOESM2_ESM.pdf]

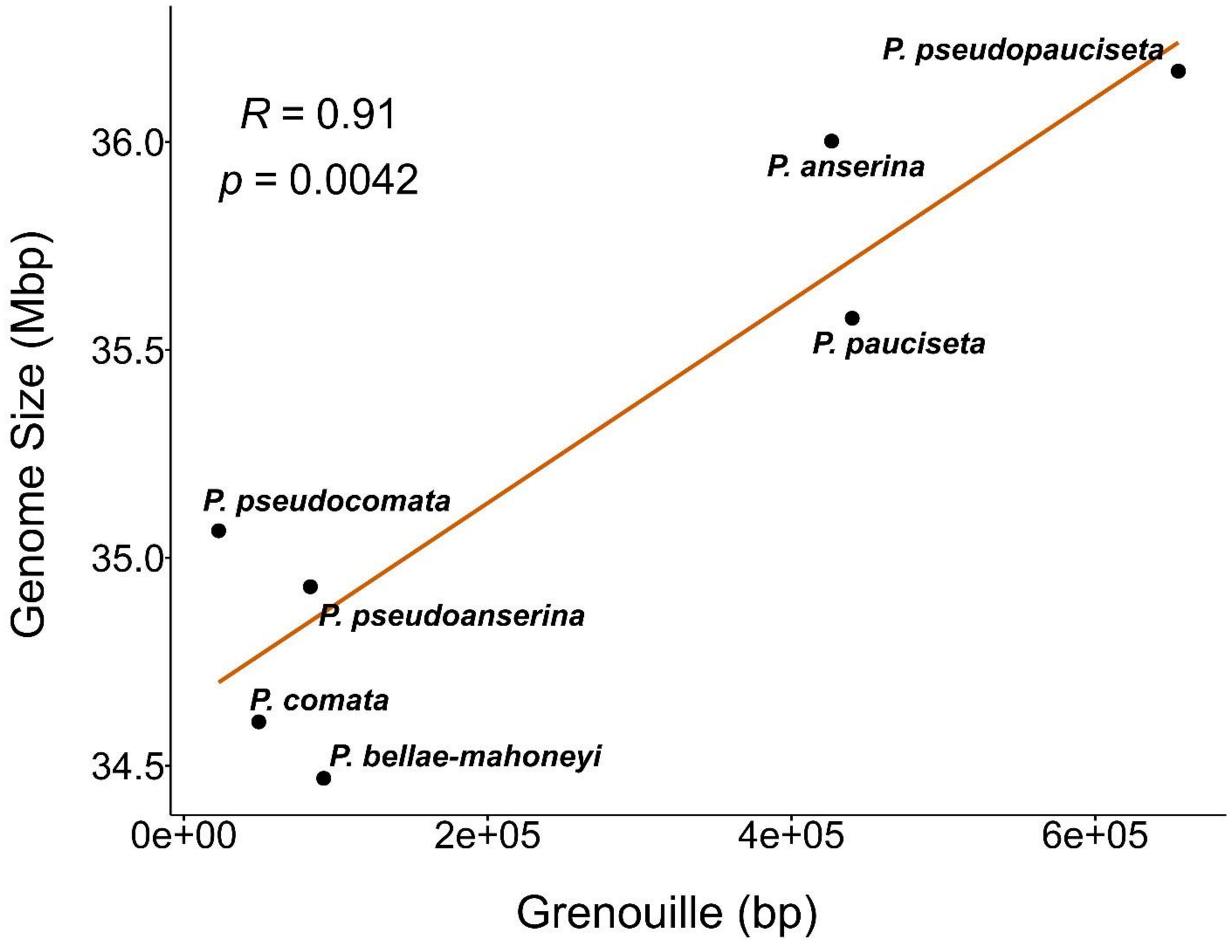

Supplementary figure 1: The LTR element grenouille abundance in base pairs is significantly associated with genome size in the species complex. Pearson´s correlation, p= 0.0042, R = 0.91.

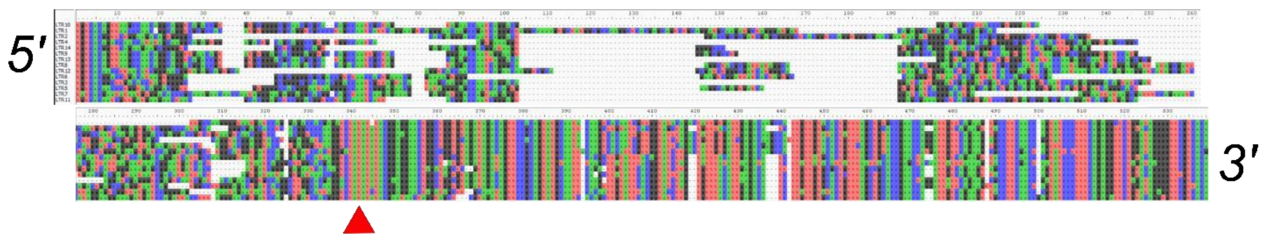

Supplementary figure 2: Alignment of Crapaud subfamily centroids. Top = 5’-half, bottom = 3’-Half. The different colors represent the nucleotides; C = blue, G = black, T = red, and A = green. The TATA-box is marked with a red arrow.

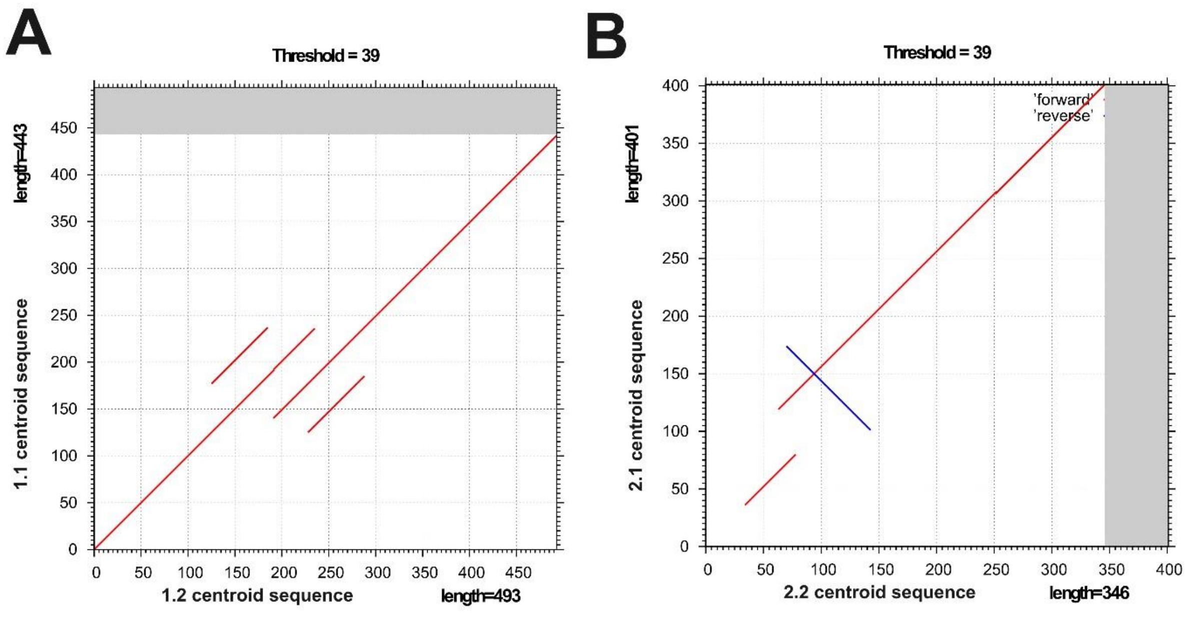

Supplementary figure 3: Dotplots between the nested cluster centroids. Red lines indicate alignments between the sequences in the forward strand. Blue lines indicate alignments in the reverse strand **A)** Community 1.1 and Community 1.2. **B)** Community 2.1 and Community 2.2.

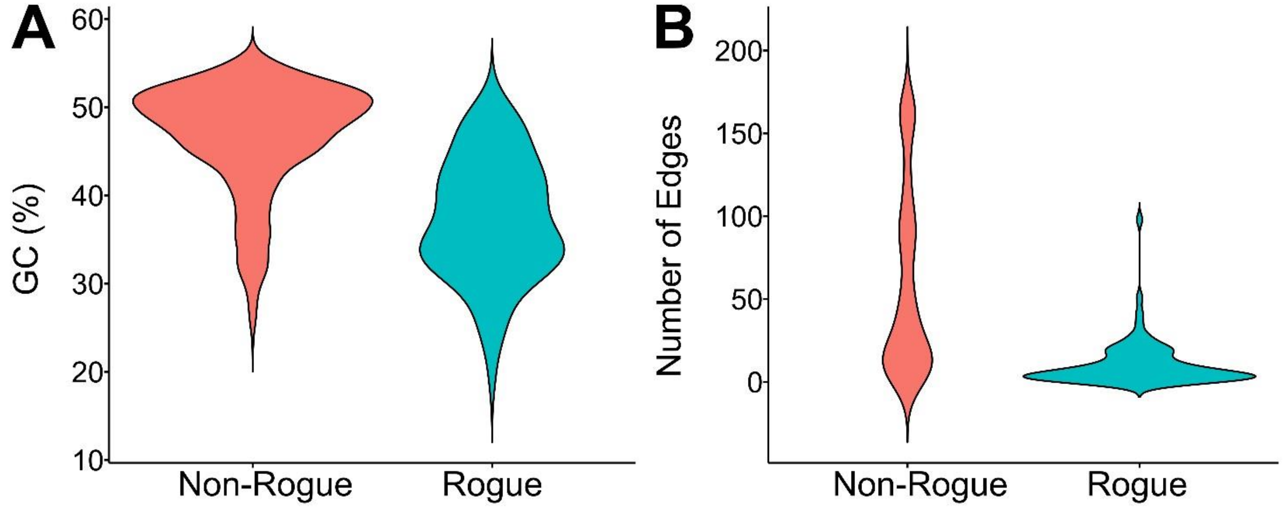

Supplementary figure 4: Comparison between classified copies inside and outside their subfamily's main clade in the ML phylogeny. **A)** GC content comparison, mean (Non-Rogue) = 48.4, mean (Rogue) = 37.4. **B)** Number of edges connected to the sequence in the SSN (Degrees), median (Non-Rogue) = 36, median (Rogue) = 6. n(Non-Rogue) = 997, n(Rogue) = 82.

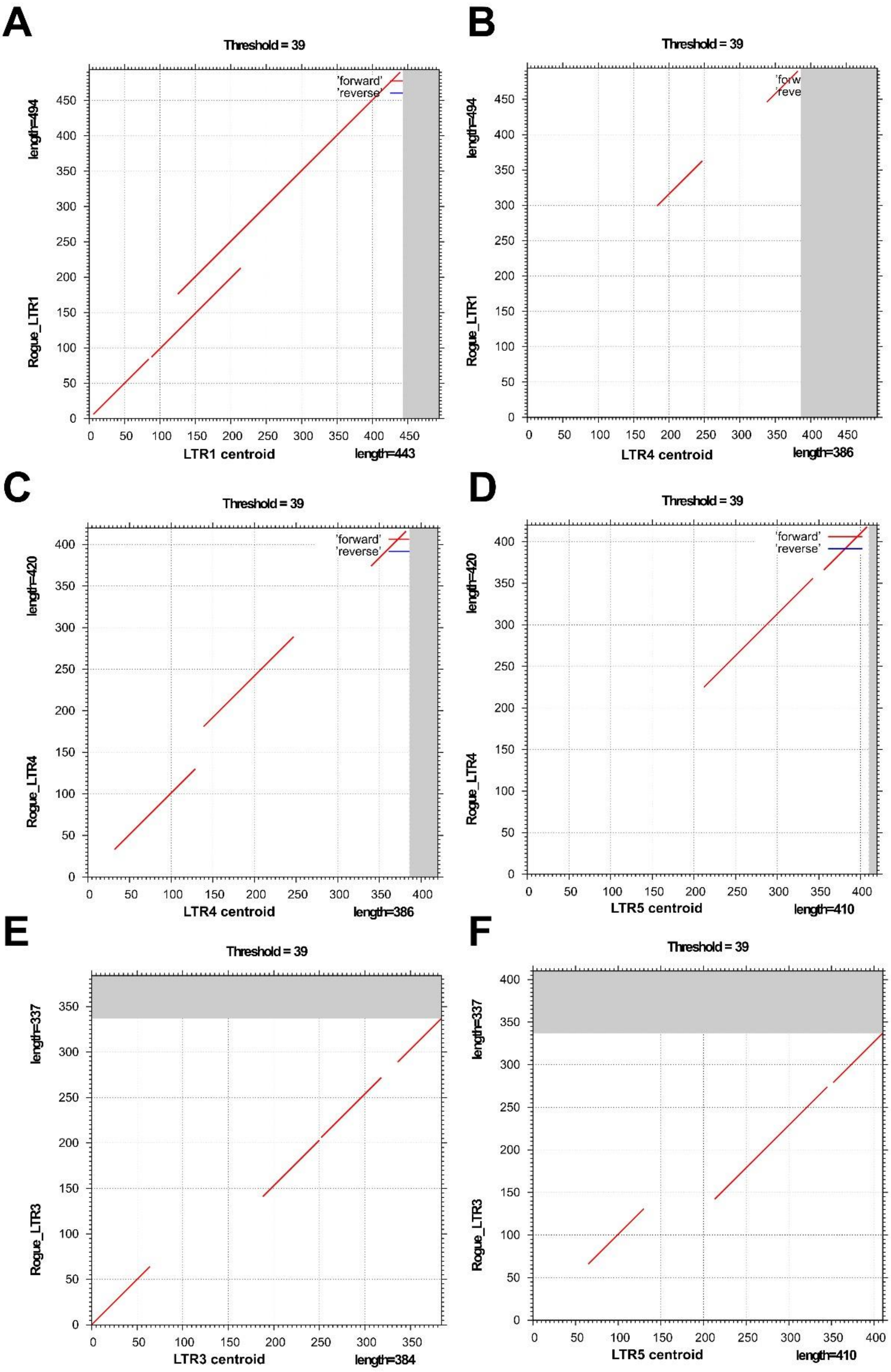

Supplementary figure 5: MAFFT alignment dotplots between representative rogue copies, the subfamily centroid, and the centroid of the clade it clusters with in the ML phylogeny. **A)** LTR1 rogue with LTR1 centroid. **B)** LTR1 rogue with LTR4 centroid. **C)** LTR4 rogue with LTR4 centroid. **D)** LTR4 rogue with LTR5 centroid. **E)** LTR3 rogue with LTR3 centroid. **F)** LTR3 rogue with LTR5 centroid

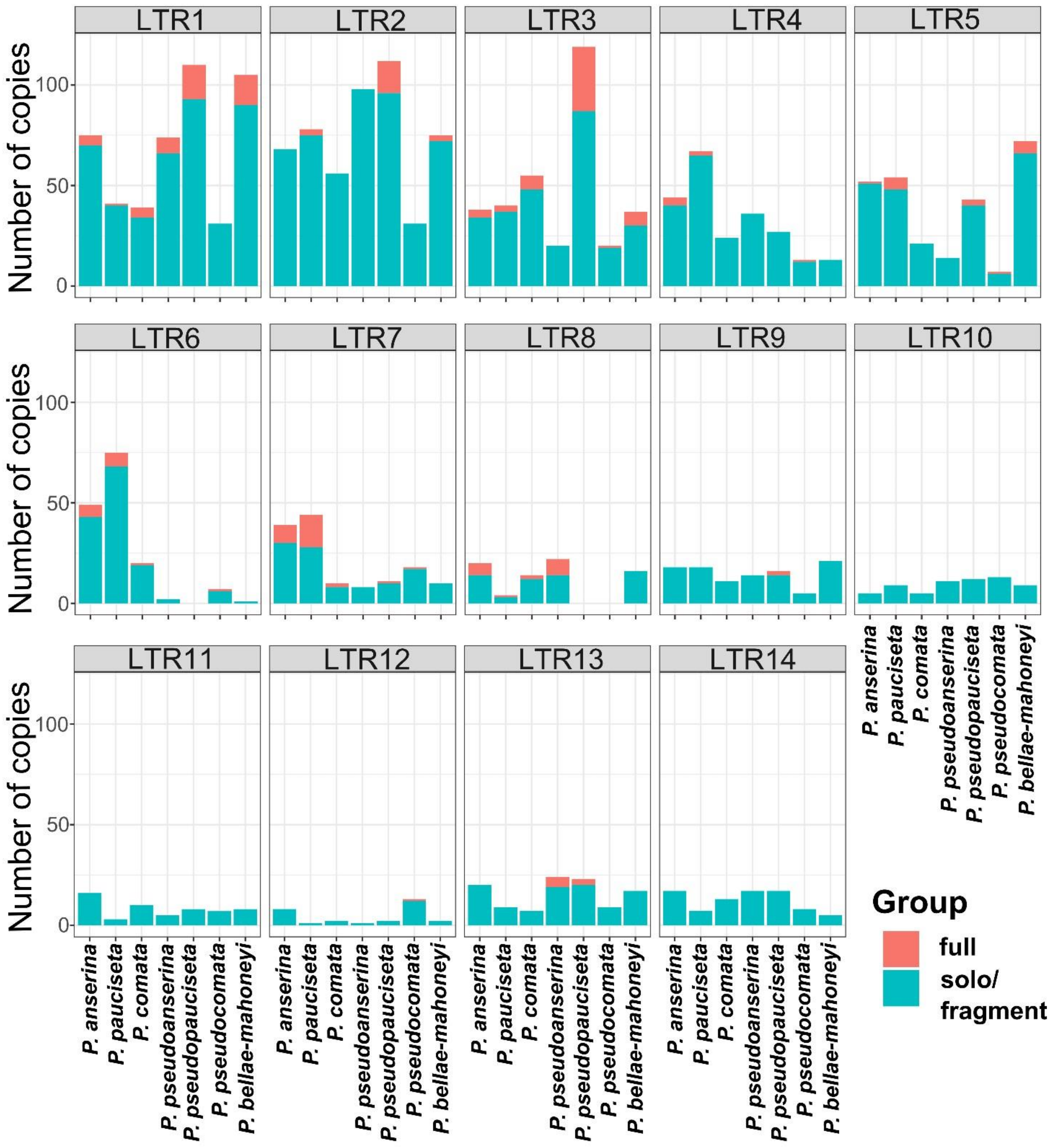

Supplementary figure 6: Number of Repeatmasker hits of LTR subfamilies in the *P. anserina* species-complex of both full copy LTRs and solo/fragment LTRs.

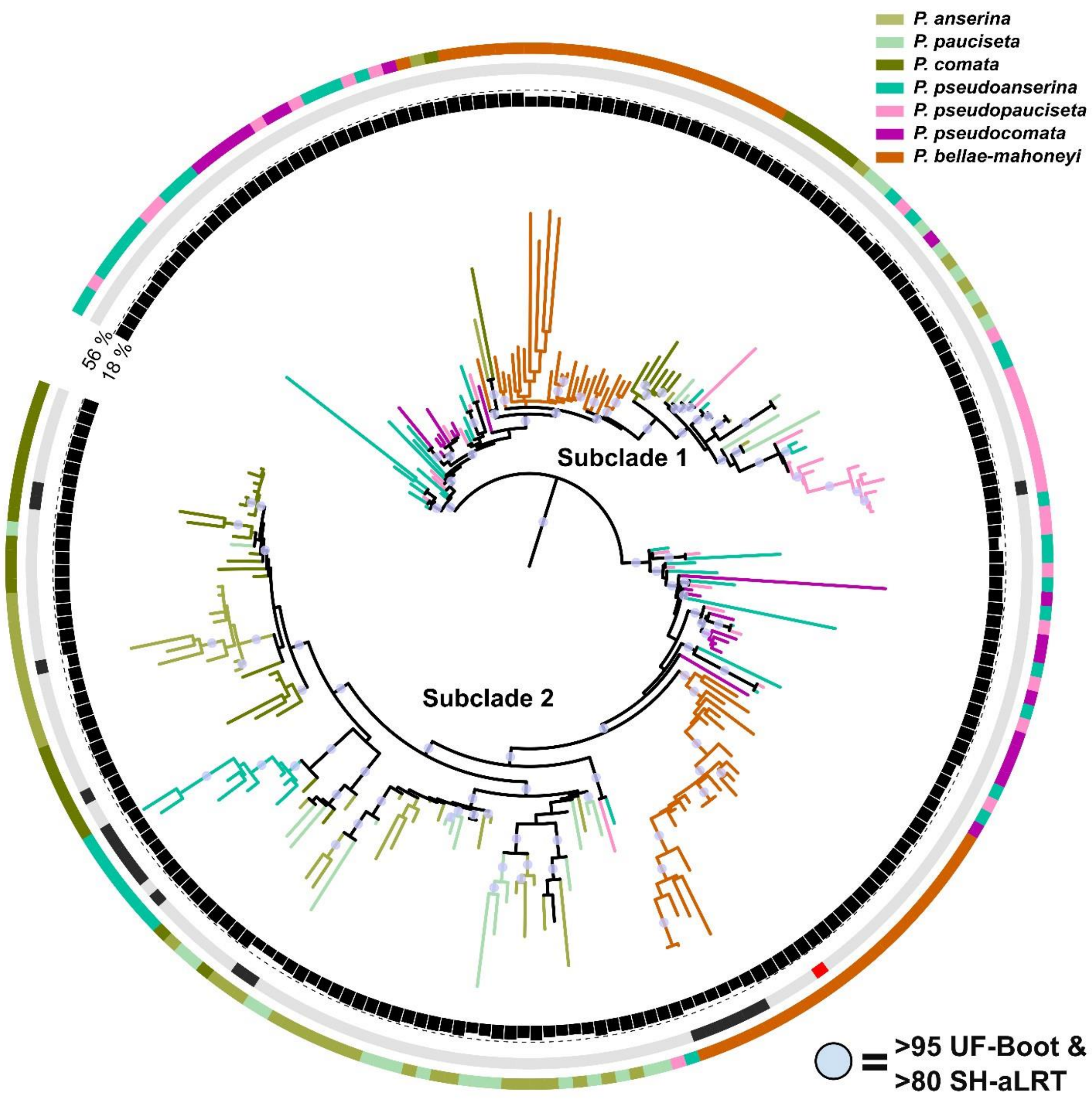

Supplementary figure 7: Pruned phylogeny of the LTR1 clade. Tracks from inner to outer: GC-content, Solo/fragment (grey) / Full (black) / Active (red), Species. Species are also indicated by colors of branches. The phylogeny was rooted based on the phylogeny including all LTR copies of the dataset.

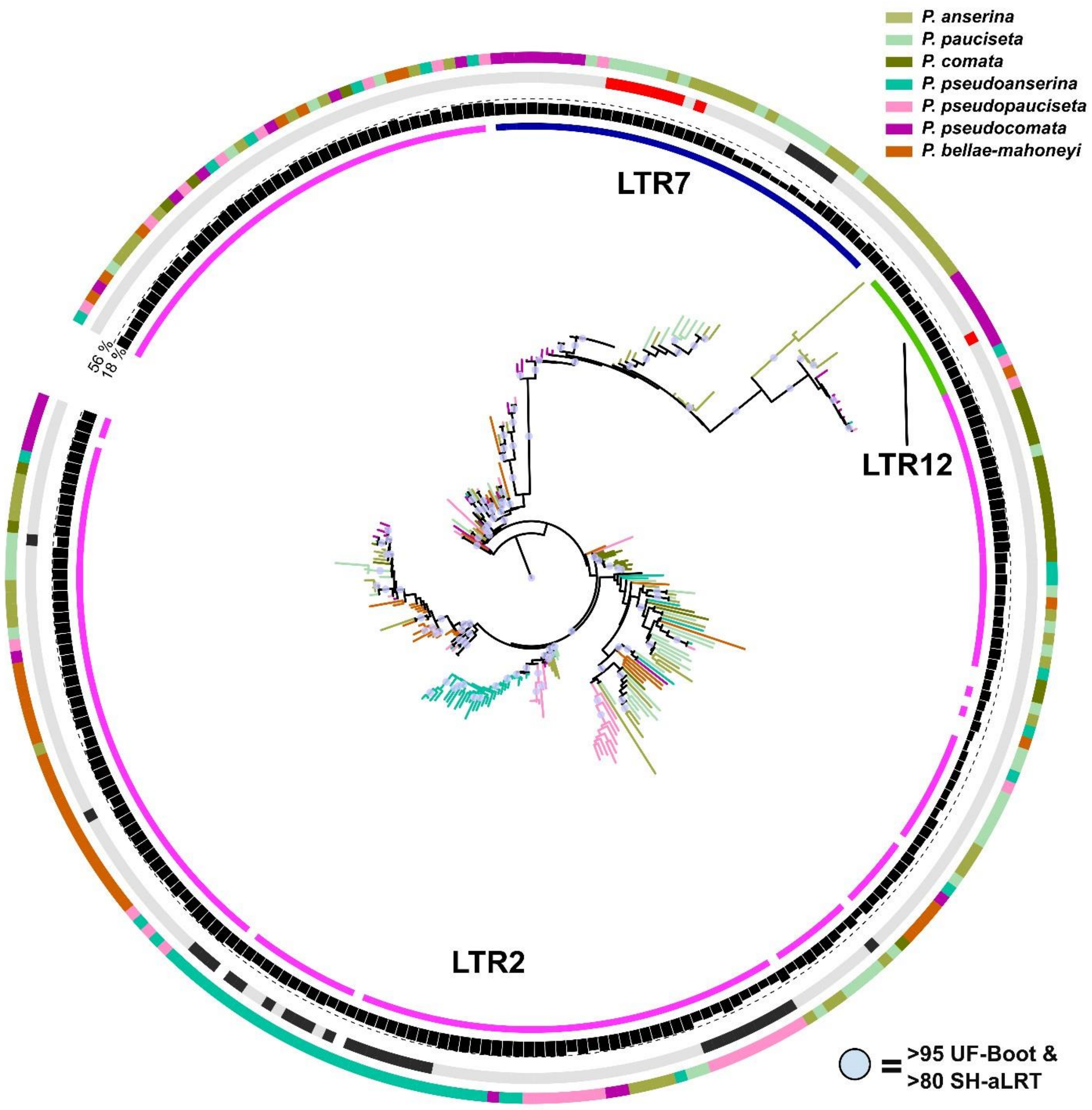

Supplementary figure 8: Pruned phylogeny of the LTR2, LTR7 and LTR12 clade. Tracks from inner to outer: GC-content, Solo/fragment (grey) / Full (black) / Active (red), Species. Species are also indicated by colors of branches. The phylogeny was rooted based on the phylogeny including all LTR copies of the dataset.

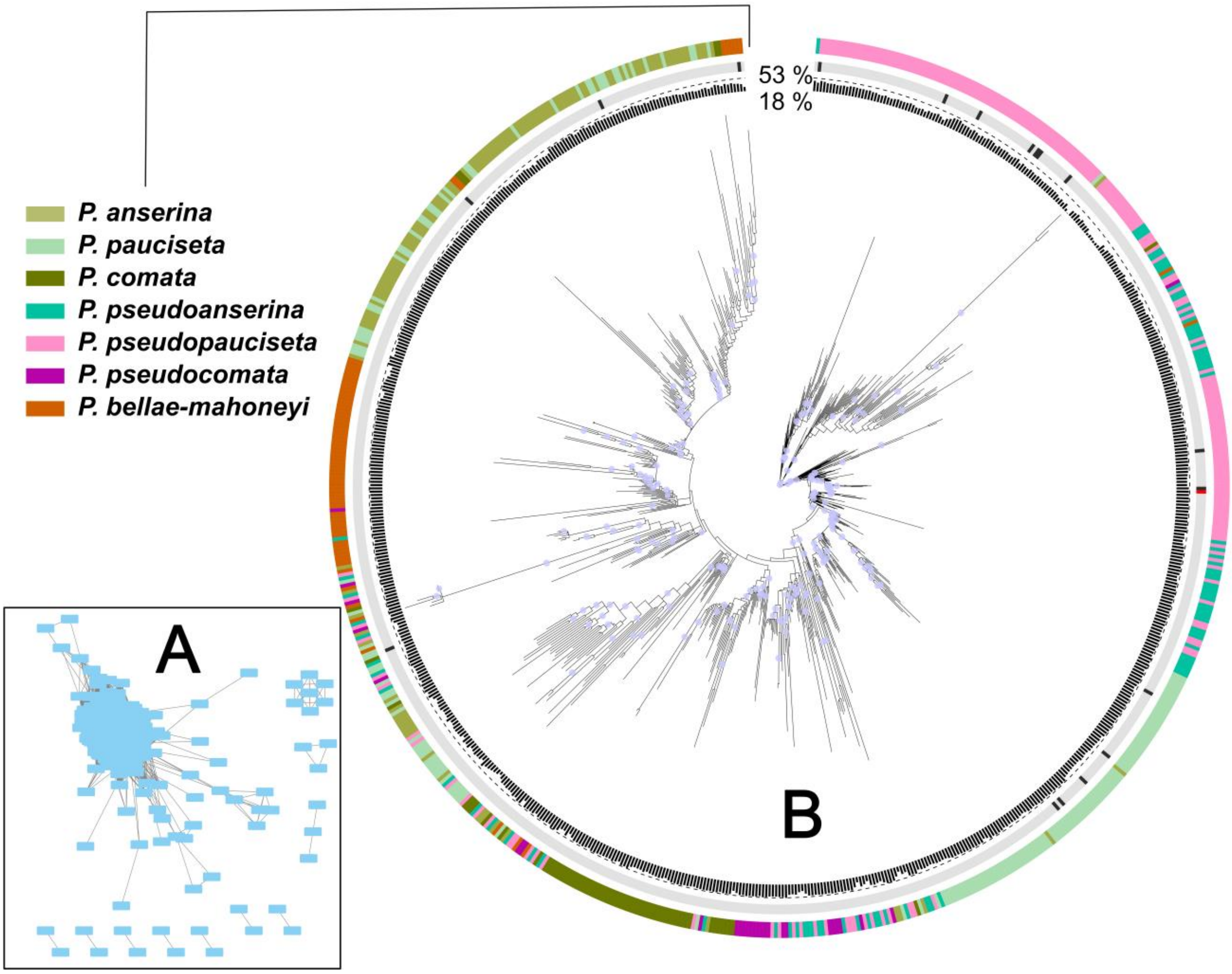

Supplementary figure 9: Analysis of the *grenouille* LTR element **A)** SSN of the *grenouille* terminal repeats using the thresholds >80% identity over >80% sequence coverage. 692 terminal repeat copies clustered in the largest network cluster. The second largest had seven copies. n= 753. **B)** ML phylogeny of the 753 terminal repeats of grenouille. Tracks from inner to outer: GC-content ranging between 18% to 53%; Full copies (black), putative active copies (red), and solo/fragment copies (grey); Species

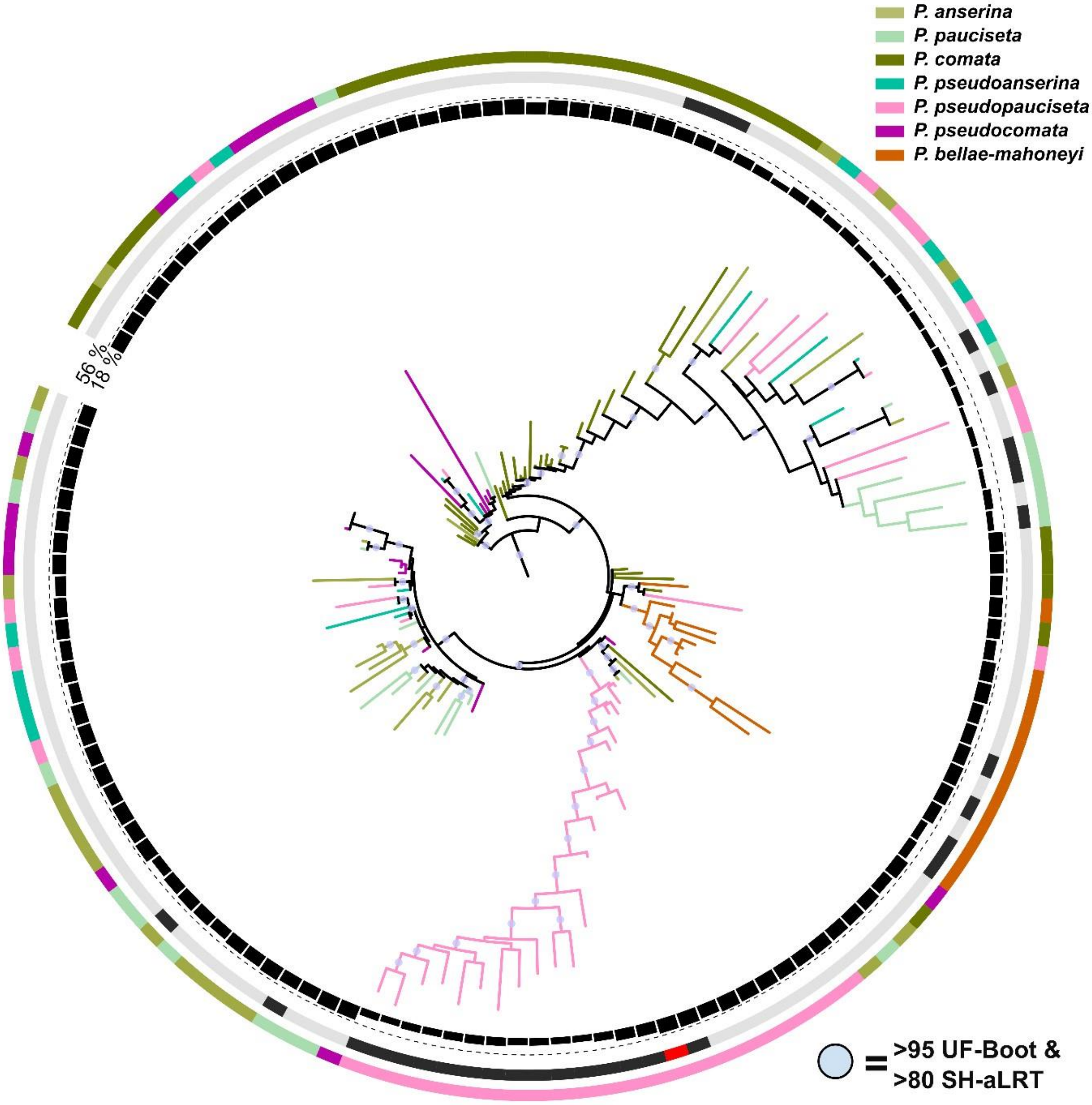

Supplementary figure 10: Pruned phylogeny of the LTR3 clade. Tracks from inner to outer: GC-content, Solo/fragment (grey) / Full (black) / Active (red), Species. Species are also indicated by colors of branches. The phylogeny was rooted based on the phylogeny including all LTR copies of the dataset.

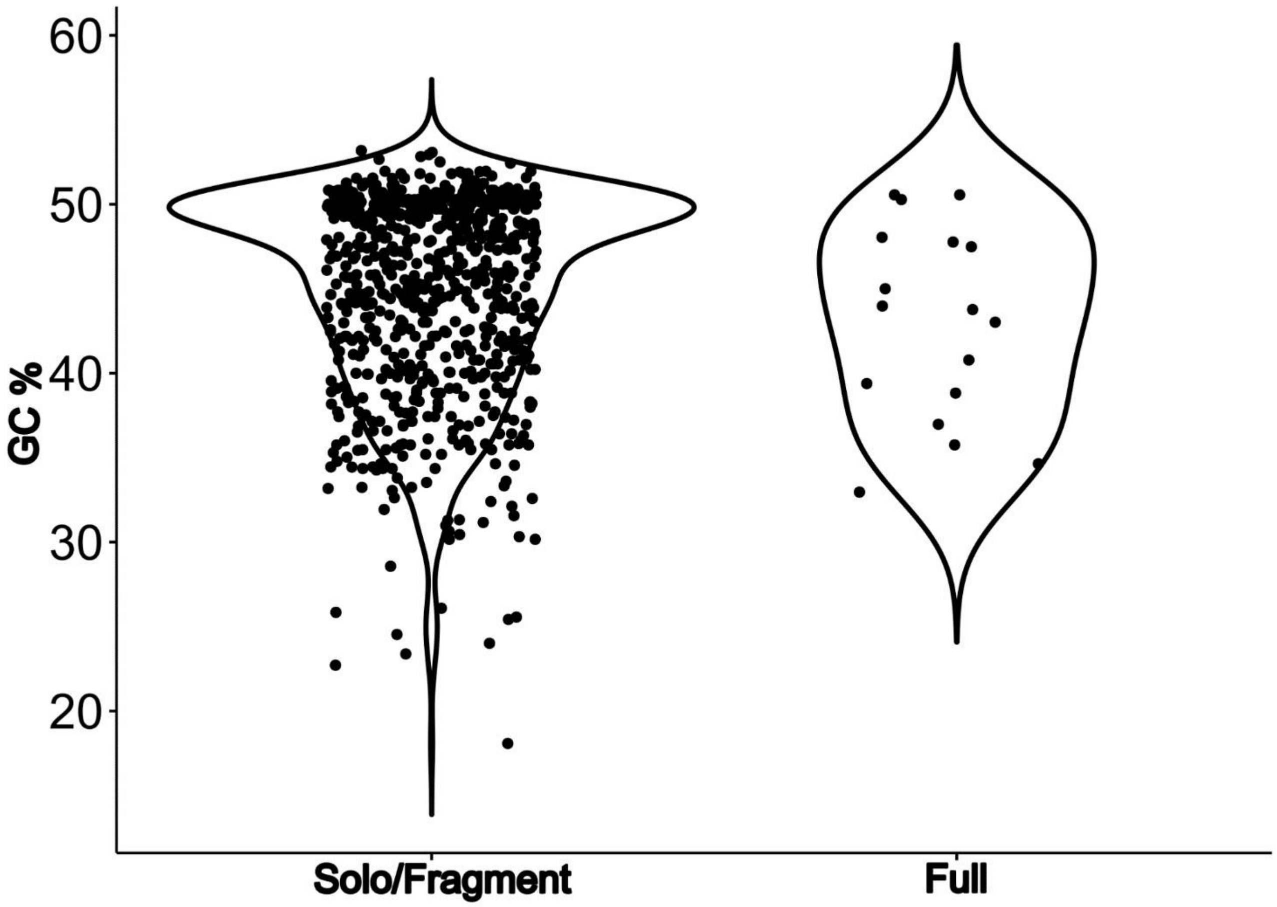

Supplementary figure 11: GC% content of the terminal repeat sequences of the *grenouille* element. divided into solo/fragment copies and full copies. n(solo/fragment) = 736, n(full) = 17.
